# PARP inhibitor-related haemorrhages: What does the real-world study say?

**DOI:** 10.3389/fonc.2023.1070343

**Published:** 2023-02-27

**Authors:** Shiyan Wang, Mengran Guo, Xiang Zhang, Ping Fan, Zhaohui Jin

**Affiliations:** ^1^ Department of Pharmacy, West China Hospital, Sichuan University, Chengdu, China; ^2^ State Key Laboratory of Biotherapy, West China Hospital, Sichuan University, Chengdu, Sichuan, China

**Keywords:** PARP inhibitors, haemorrhage, FAERS database, pharmacovigilance, niraparib

## Abstract

**Background:**

PARP inhibitors (PARPis) are novel molecular targeted therapeutics for inhibition of DNA repair in tumor cells, which are commonly used in ovarian cancer. Recent case reports have indicated that haemorrhages-related adverse events may be associated with PARPis. However, little is known about the characteristics and signal strength factors of this kind of adverse event.

**Methods:**

A pharmacovigilance study from January 2004 to March 2022 based on the FDA adverse event reporting system (FAERS) database was conducted by adopting the proportional imbalance method based on the four algorithms, including the reporting odds ratio (ROR), proportional reporting ratio (PRR), Bayesian confidence propagation neural networks (BCPNN) and multi-item gamma Poisson shrinker (MGPS).

**Results:**

725 cases of PARPi-haemorrhages-related adverse events were identified with a fatality rate of 4.72% (30/725) and a median age of 67 years. About 88% of the adverse events occurred within 6 months, and the median duration (IQR) was 68 days. Most adverse events (n=477, 75.11%) were related to the treatment of niraparib. Importantly, niraparib exposure was associated with a significant increase in haemorrhages-related adverse events (ROR (95% CI), 1.13(1.03,1.23), PRR (χ2), 1.12(7.32), IC (IC 025), 0.17(0.15). In addition, petechiae, gingival bleeding, bloody urine, as well as rectal haemorrhage should be monitored when using niraparib.

**Conclusion:**

Recognition and management of PARPi-haemorrhages-related adverse events is of significance to clinical practice. In this study, we provided a safety signal that haemorrhage-related adverse events should be monitored for when using niraparib. However, larger and more robust post-market safety studies are needed to improve the quality of this evidence.

## 1 Introduction

Poly (ADP-ribose) polymerase (PARP) is an important factor mediating DNA repair, which can be combined with other DNA repair proteins to repair DNA damage (1). PARP inhibitors (PARPis) are one of the most promising PARP-targeted drugs for some cancers, such as ovarian cancer, breast cancer, pancreatic cancer, and prostate cancer (2–5).

In 2014, the world’s first PARPi, olaparib, was approved by the U.S. Food and Drug Administration (FDA) to treat breast, ovarian, pancreatic, and prostate cancers (6, 7). Thereafter, rucaparib and niraparib appeared successively in 2016 and 2017 (8). Up to now, together with talazoparib, four PARP-targeted drugs have been approved by the FDA for marketing. Their widespread use, however, induces some drug-related adverse reactions. The adverse reactions of PARPis include haematological toxicity (9, 10), gastrointestinal toxicity (11), etc., and each drug also has its specific toxicities. As summarized in **Table 1** (12–16), anemia, fatigue/asthenia, and nausea are three common adverse reactions for all PARPis. For niraparib, thrombocytopenia is a frequent adverse event for which the proportion of any grade was 61.3% and grade ≥3 was 33.8% (13). Other studies also noted the association between talazoparib and frequent grade ≥3 hematologic adverse events (anemia 39%, neutropenia 21%, and thrombocytopenia 15%) (17). Gastrointestinal toxicities are also common adverse reactions for all PARPis, as side effects of nausea were reported in 152 (77.7%) of 260 patients treated with olaparib (12), 270 (74%) of 367 patients treated with niraparib (13), and 280 (75%) of 372 patients treated with rucaparib (14). Recently, one death due to gastric haemorrhage has been reported in a patient using niraparib monotherapy (18). This indicates that haemorrhage may be a serious but non-negligible adverse reaction for PARPis. However, individual case reports may be insufficient to assess the association between PARPi therapy and this rare adverse effect. The real-world evidence remains limited. Furthermore, it is unclear whether the haemorrhages-related adverse event happens after administration of other PARPis. Therefore, we conducted a disproportionality analysis by using the FAERS database to characterize and evaluate haemorrhages-related adverse events associated with PARPis.

**Table 1:** The most common adverse events and their percentages related to each PARP inhibitor.

Patient With AE	Olaparib (N=260) (%)		Niraparib (N=367) (%)		Rucaparib (N=372) (%)		Talazoparib (N=127) (%)		Veliparib (N=376) (%)	
	Any Grade	Grade ≥3	Any Grade	Grade ≥3	Any Grade	Grade ≥3	Any Grade	Grade ≥3	Any Grade	Grade ≥3
Blood and lymphatic system disorders										
Anemia (Includes patients with anemia or decreased hemoglobin)	40.0	21.9	50.1	25.3	37.0	19.0	49.0	31.0	65.0	41.0
Leukopenia	/	/	/	/	/	/	10.0	1.0	23.0	12.0
Lymphopenia	/	/	/	/	/	/	8.0	5.0	/	/
Neutropenia (Includes patients with neutropenia, febrile neutropenia, or decreased neutrophil count.)	23.1	8.5	30.2	19.6	18.0	7.0	17.0	8.0	75.0	62.0
Thrombocytopenia (Includes patients with thrombocytopenia or decreased platelet count)	11.2	0.8	61.3	33.8	28.0	5.0	19.0	9.0	60.0	31.0
Gastrointestinal disorders										
Abdominal distention	/	/	7.6	0.0	11.0	0.0	/	/	/	/
Abdominal pain	25.8	1.5	22.6	1.1	30.0	2.0	4.0	1.0	/	/
Upper abdominal pain	17.3	0.0	/	/	14.0	1.0	/	/	/	/
Constipation	27.7	0.0	39.8	0.5	37.0	2.0	18.0	1.0	48.0	2.0
Diarrhea	34.6	3.1	19.1	0.3	32.0	1.0	17.0	0.0	37.0	3.0
Dyspepsia	16.5	0.0	11.4	0.0	15.0	<1	/	/	/	/
Nausea	77.7	0.8	73.6	3.0	75.0	4.0	33.0	2.0	72.0	4.0
Vomiting	40.0	0.4	34.3	1.9	37.0	4.0	14.0	2.0	35.0	4.0
General disorders and administration site conditions										
Alopecia	/	/	/	/	/	/	/	/	57.0	0.0
Fatigue or asthenia	64.2	3.8	59.4	8.2	69.0	7.0	44.0	6.0	62.0	5.0
Hypertension			19.3	8.2	/	/	5.0	1.0	/	/
Nasopharyngitis	10.8	0.0	11.2	0.0	/	/	/	/	/	/
Palpitations	/	/	10.4	0.0	/	/	/	/	/	/
Peripheral oedema	/	/	/	/	10.0	<1	/	/	/	/
Peripheral sensory neuropathy	/	/	/	/	/	/	/	/	63.0	2.0
Pyrexia	12.3	0.0	/	/	12.0	0.0	/	/	/	/
Infections and infestations										
Upper respiratory tract infection	11.5	0.0	/	/	11.0	0.0	/	/	/	/
Urinary tract infection	11.9	0.8	10.4	0.8			8.0	2.0	/	/
Investigations										
Increase in alanine aminotransferase or aspartate aminotransferase concentration	/	/	/	/	34.0	10.0	16.0	4.0	/	/
Increase in blood creatinine concentration	/	/	/	/	15.0	<1	/	/	/	/
Metabolism and nutrition disorders										
Decreased appetite	20.4	0.0	25.3	0.3	23.0	1.0	28.0	3.0	22.0	1.0
Hypomagnesaemia	/	/	/	/	11.0	<1	/	/	25.0	1.0
Musculoskeletal and connective tissue disorders										
Arthralgia	28.8	0.0	11.7	0.3	15.0	1.0	8.0	1.0	28.0	<1
Back pain	16.2	0.0	13.4	0.5	12.0	0.0	/	/	/	/
Bone pain	/	/	/	/	/	/	7.0	1.0	/	/
Myalgia	10.0	0.0	8.2	0.3	/	/	/	/	/	/
Musculoskeletal pain	/	/	/	/	/	/	7.0	2.0	/	/
Pain in extremity	11.5	0.0	/	/	/	/	10.0	2.0	/	/
Nervous system disorders										
Dysgeusia	21.5	0.0	10.1	0.0	39.0	0.0	/	/	/	/
Dizziness	20.4	0.0	16.6	0.0	15.0	0.0	12.0	0.0	22.0	1.0
Depression	5.4	0.4	/	/	/	/	/	/	/	/
Headache	23.1	0.4	25.9	0.3	18.0	<1	7.0	1.0	24.0	1.0
Paraesthesia	/	/	/	/	/	/	7.0	1.0	/	/
Psychiatric disorders										
Insomnia	10.4	0.0	24.3	0.3	14.0	0.0	/	/	32.0	<1
Respiratory, thoracic, and mediastinal disorders										
Cough	16.9	0.0	15.0	0.0	15.0	0.0	/	/	/	/
Dyspnea	15.8	0.0	19.3	1.1	13.0	0.0	14.0	2.0	24.0	2.0
Pulmonary embolism	/	/	/	/	/	/	6.0	5.0	/	/
Skin and subcutaneous tissue disorders										
Photosensitivity reaction	/	/	/	/	17.0	1.0	/	/	/	/
Pruritus	/	/	/	/	13.0	0.0	/	/	/	/
Rash	/	/	/	/	12.0	<1	/	/	/	/
Urinary system										
Dysuria	/	/	/	/	/	/	6.0	1.0	/	/
Haematuria	/	/	/	/	/	/	7.0	1.0	/	/

## 2 Article types

Original Research Articles

## 3 Methods

### 3.1 Data source

We conducted a pharmacovigilance study based on the FAERS database. The FAERS database collects adverse drug event (ADEs) reports by consumers, health professionals, pharmaceutical manufacturers, and patients from different regions. FAERS data are available to the public. The FAERS data include demographic and administrative information, drug information, reaction information, patient outcomes, the source of the report, therapy start dates and end dates for reported drugs, and indications for use. The data were collected from 2004 Quarter 1 (Q1) to 2022 Quarter 1 (Q1) in the FAERS database for this study.

### 3.2 Data mapping

PARPis studied on the market include olaparib (Lynparza), niraparib (Zejula), rucaparib (Rubraca), talazoparib (Talzenna) and veliparib (in Phase III clinical trials). The reports of the FAERS database were coded using MedDRA (V25.0) preferred terms (PTs)-related to haemorrhages-related adverse events: “haemorrhage [10055798]”, “bleeding [10005103]”, “peptic ulcer [10034341]”, “intracranial haemorrhage [10018985]”, “thrombocytopenia [10043554]”, “hemolytic anemia [10018916]”, “purpura [10037549]”, “epistaxis [10015090]”, “gastrointestinal haemorrhage [10017955]”, “bruising [10006504]”.

### 3.3 Data mining

In this study, all data mining and statistical analyses were performed using SAS software (ver. 9.4; SAS Institute Inc., Cary, NC). We adopted reporting odds ratio (ROR), proportional reporting ratio (PRR), the Bayesian confidence propagation neural networks (BCPNN) and the multi-item gamma Poisson shrinker (MGPS) algorithms to investigate the associations between haemorrhage and PARPis. This proportional imbalance method is based on the above four algorithms. This method (19–27) compares the proportion of a certain event of the target drug in the ADE spontaneous reporting system with the proportion of the target event of all other drugs (background data). Statistical associations between this target drug and events will be investigated to detect potential ADE signals. The frequency and signal strength between the target drug and the adverse event greater than the threshold indicates disproportionality, and one signal is prompted to generate. When both the number of co-occurrences (N) ≥2 and the lower limit of 95% CI of ROR > 1 are satisfied, a signal is indicated. When both PRR ≥ 2 and the chi-squared (χ^2)^ ≥ 4 and the number of co-occurrences ≥ 3 are satisfied, a signal is indicated. When the lower limit of the 95% two-sided CI of the IC (IC025) > 0 is satisfied, a signal is indicated. When both the number of co-occurrences > 0 and the lower 90% one-sided CI of EBGM (EB05) ≥ 2, a signal is indicated.

## 4 Results

### 4.1 Basic information of the patients

A total of 725 cases of PARPi-associated haemorrhages adverse events were identified from the database. We collected their clinical characteristics (age, gender, reporters, reporting region and reporting year) (**Table 2**) and excluded the incorrect or blank records. The number of PARPi-haemorrhages-related adverse events reports increased dramatically from 2016 to 2022 (3 cases in 2015, 120 cases in 2019, 126 cases in 2020, 177 cases in 2021, and 60 cases in 2022 Quarter 1). As shown in **Table 2**, a majority of cases originated from America (84.83%) and most patients were middle-aged adults, with a median [interquartile range (IQR)] age of 67 years. Patients aged 18-44 and older than 85 took up a small proportion. In addition, the number of female (*n*=574, 79.17%) patients differed greatly to that of male patients (*n*=30, 4.12%), because the indication is mainly ovarian cancer. For talazoparib and veliparib, only female patients experienced heamorrhages-related adverse events. Of the cases reported, 468 were by consumers (64.55%). Niraparib, the third PARPi on the market, was associated with the most cases of adverse events (n=477, 75.11%).

**Table 2:** Characteristics of patients with haemorrhages-related adverse events associated with different PARPis.

Characteristics	Olaparib	Niraparib	Rucaparib	Talazoparib	Veliparib	Total
Age						
18-44	5	5	3	3	1	17
45-64	22	95	36	3	1	157
65-74	20	87	22			129
75-84	8	50	7			65
≥85		5				5
Missing	33	275	44			352
Gender						
Female	68	395	103	6	2	574
Male	16	9	5			30
Missing	4	113	4			121
Reporters						
Consumer	18	405	43	2		468
Other health-professional	4	25	34		1	64
Pharmacist	1	7	2			10
Physician	32	61	14	2	1	110
Missing	33	19	19	2		73
Reporting region						
Africa		1				1
Asian	25	23		2		50
Europe	11	34	3	2		50
North America	47	456	109	1	2	615
Oceania	1	1				2
South America	4	2		1		7
Reporting year						
2015	1				2	3
2016	5					5
2017	10	40	14			64
2018	7	143	20			170
2019	19	73	26	2		120
2020	15	80	29	2		126
2021	25	127	23	2		177
2022	6	54				60

### 4.2 Indications for using PARPis

The indications for using PARPis are shown in **Table 3**. Patients who received PARPis mainly had ovarian cancer (527/725, 72.69%), malignant peritoneal neoplasm (32/725, 4.41%), fallopian tube cancer (29/725, 4.00%), prostate cancer (12/725, 1.66%), breast cancer (10/725, 1.38%), pancreatic carcinoma (7/725, 0.97%), endometrial cancer (6/725, 0.83%) or other cancers (77/725, 10.62%). For each PARPi, olaparib, niraparib, rucaparib and veliparib were mainly used in patients with ovarian cancer. Olaparib, niraparib, and rucaparib were also used in patients with malignant peritoneal neoplasm, fallopian tube cancer, breast cancer, and prostate cancer, while talazoparib was used mainly in breast cancer patients.

**Table 3:** Indications for the use of the five PARPis.

Indication	Reports (N, %)					
	Olaparib	Niraparib	Rucaparib	Talazoparib	Veliparib	Total
Ovarian cancer	52(7.27)	387(53.38)	87(12.00)		1(0.14)	527(72.69)
Malignant peritoneal neoplasm	2(0.28)	28(3.86)	2(0.28)			32(4.41)
Fallopian tube cancer	3(0.41)	22(3.03)	4(0.55)			29(4.00)
Breast cancer	5(0.69)	1(0.14)	1(0.14)	3(0.41)		10(1.38)
Prostate cancer	4(0.55)	3(0.41)	5(0.69)			12(1.66)
Pancreatic carcinoma	5(0.69)	2(0.28)				7(0.97)
Endometrial cancer		5(0.69)	1(0.14)			6(0.83)
Other cancers	9(1.24)	54(7.45)	12(1.66)	2(0.28)		77(10.62)
Missing	8(1.1)	15(2.07)		1(0.14)	1(0.14)	25(3.45)

### 4.3 Onset times of haemorrhages

**Figure 1:**
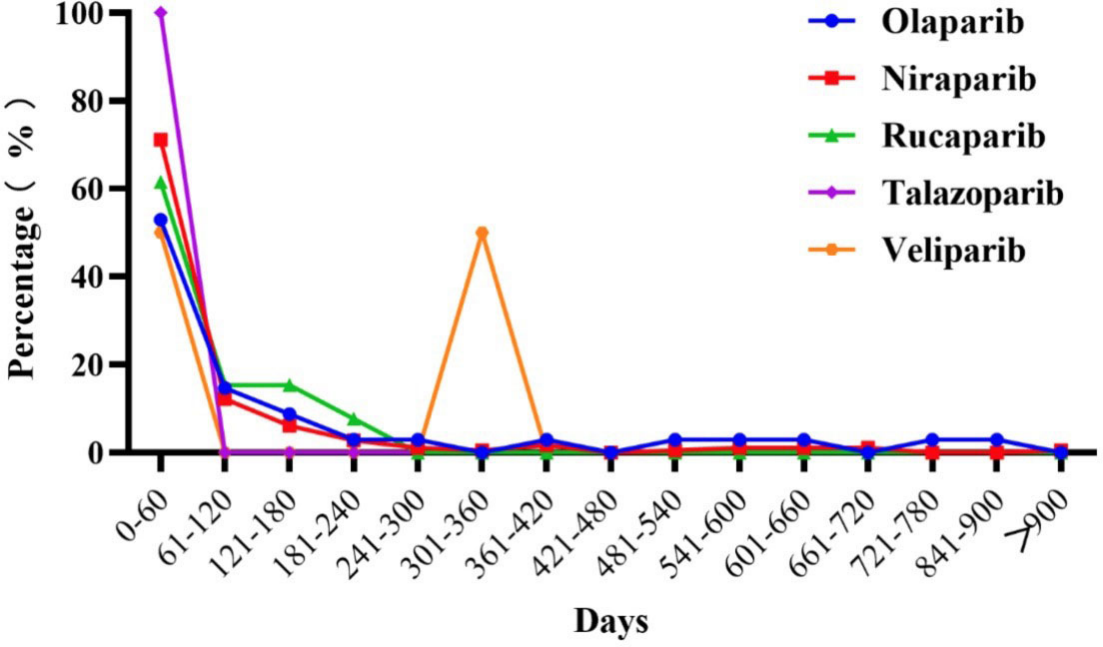
The proportion of onset time of PARPis haemorrhages-related adverse reactions.

The onset time was defined as from the start date of the PARPis administration to the heamorrhages-related adverse events onset date. The proportions of onset time of PARPis haemorrhages-related adverse reactions are shown in **Figure 1**. The median duration (IQR) was 68 (IQR: 9-77) days and the duration ranged from 1 day to 1530 days. About 88% of the adverse events occurred within 6 months. Overall, the median onset times of olaparib, niraparib, rucaparib, talazoparib were 158 (IQR: 6-164) days, 64.25 (IQR: 9-73.25) days, 70 (IQR: 0-70) days, and 20.5 (IQR: 13.5-34) days, respectively. Veliparib had only 2 cases that the haemorrhages-related adverse events onset date was 46 days and 322 days, respectively.

### 4.4 Detailed PT related to haemorrhages-related adverse events

A total of 68 PTs (725 cases) related to PARPis heamorrhages-related adverse events were screened out. The detailed PTs for using PARPis are shown in **Table 4**. The top 10 PTs are haemorrhage (108, 14.90%), petechiae (90, 12.41%), gingival bleeding (75, 10.34%), blood urine present (62, 8.55%), rectal haemorrhage (55, 7.59%), vaginal haemorrhage (46, 6.34%), gastrointestinal haemorrhage (30, 4.14%), cerebral haemorrhage (25, 3.45%), mouth haemorrhage (25, 3.45%), eye haemorrhage (14, 1.93%), and skin haemorrhage (14, 1.93%), respectively. Among these PTs, there were 108 “haemorrhage” cases that did not identify the site of bleeding. For olaparib, there are 33 PTs (81 cases) associated heamorrhages-related adverse events. Except for “haemorrhage” PT, gastrointestinal haemorrhage (13,16.05%), rectal haemorrhage (9, 11.11%), cerebral haemorrhage (6, 7.41%), and vaginal haemorrhage (5, 6.17%) had the highest frequencies. Niraparib has 55 PTs (517 cases) associated heamorrhages-related adverse events. Apart from “haemorrhage”, niraparib was more likely to induce petechiae (81, 15.67%), gingival bleeding (58, 11.22%), blood urine (39, 7.54%), as well as rectal haemorrhage (38, 7.35%). Rucaparib exhibited higher haemorrhages-related adverse event frequency of bloody urine (22, 19.13%), vaginal haemorrhage (15, 13.04%), and gingival bleeding (13, 11.30%). As for talazoparib, there were 4 cases (40%) of gingival bleeding. Haemorrhage intracranial and lower gastrointestinal haemorrhage are the 2 PTs with veliparib.

**Table 4:** Detailed PT-related to haemorrhages-related adverse events associated with different PARPis.

PT	Reports (N, %)					
	Olaparib	Niraparib	Rucaparib	Talazoparib	Veliparib	Total
Haemorrhage	12 (14.81)	83 (16.05)	12 (10.43)	1 (10)		108 (14.90)
Petechiae	2 (2.47)	81 (15.67)	6 (5.22)	1 (10)		90 (12.41)
Gingival bleeding		58 (11.22)	13 (11.30)	4 (40)		75 (10.34)
Blood urine present	1 (1.23)	39 (7.54)	22 (19.13)			62 (8.55)
Rectal haemorrhage	9 (11.11)	38 (7.35)	8 (6.96)			55 (7.59)
Vaginal haemorrhage	5 (6.17)	25 (4.84)	15 (13.04)	1(10)		46 (6.34)
Gastrointestinal haemorrhage	13 (16.05)	16 (3.09)	1 (0.87)			30 (4.14)
Cerebral haemorrhage	6 (7.41)	18 (3.48)	1 (0.87)			25 (3.45)
Mouth haemorrhage	1 (1.23)	21 (4.06)	3 (2.61)			25 (3.45)
Eye haemorrhage		13 (2.51)	1 (0.87)			14 (1.93)
Skin haemorrhage	1 (1.23)	6 (1.16)	6 (5.22)	1 (10)		14 (1.93)
Internal haemorrhage		10 (1.93)	3 (2.61)			13 (1.79)
Haemorrhage urinary tract	2 (2.47)	7 (1.35)	2 (1.74)			11 (1.52)
Tumor haemorrhage		7 (1.35)	3 (2.61)			10 (1.38)
Stoma site haemorrhage	1 (1.23)	5 (0.97)	3 (2.61)			9 (1.24)
Haemorrhage subcutaneous	1 (1.23)	7 (1.35)				8 (1.10)
Haemorrhage intracranial	1 (1.23)	3 (0.58)	1 (0.87)	1 (10)	1 (50)	7 (0.97)
Gastric haemorrhage	1 (1.23)	4 (0.77)	1 (0.87)			6 (0.83)
Haemorrhoidal haemorrhage		6 (1.16)				6 (0.83)
Ecchymosis	2 (2.47)	4 (0.77)				6 (0.83)
Wound haemorrhage		4 (0.77)	2 (1.74)			6 (0.83)
Bleeding time prolonged	1 (1.23)	3 (0.58)	2 (1.74)			6 (0.83)
Lower gastrointestinal haemorrhage	2 (2.47)	2 (0.39)			1 (50)	5 (0.69)
Lip haemorrhage	1 (1.23)	2 (0.39)	2 (1.74)			5 (0.69)
Pharyngeal haemorrhage		5 (0.97)				5 (0.69)
Purpura		5 (0.97)				5 (0.69)
Conjunctival haemorrhage		3 (0.58)	1 (0.87)			4 (0.55)
Urinary bladder haemorrhage		4 (0.77)				4 (0.55)
Immune thrombocytopenic purpura	1 (1.23)	3 (0.58)				4 (0.55)
Subarachnoid haemorrhage	2 (2.47)	1 (0.19)				3 (0.41)
Intestinal haemorrhage		3 (0.58)				3 (0.41)
Large intestinal haemorrhage		3 (0.58)				3 (0.41)
Thrombocytopenic purpura	1 (1.23)	2 (0.39)				3 (0.41)
Thrombotic thrombocytopenic purpura	2 (2.47)	1 (0.19)				3 (0.41)
Post procedural haemorrhage		2 (0.39)	1 (0.87)			3 (0.41)
Upper gastrointestinal haemorrhage		1 (0.19)	1 (0.87)			2 (0.28)
Ulcer haemorrhage	1 (1.23)	1 (0.19)				2 (0.28)
Anal fissure haemorrhage	2 (2.47)					2 (0.28)
Purpura senile		2 (0.39)				2 (0.28)
Injection site haemorrhage		2 (0.39)				2 (0.28)
Intermenstrual bleeding		1 (0.19)		1 (10)		2 (0.28)
Intra-abdominal haemorrhageI	2 (2.47)					2 (0.28)
Oesophageal haemorrhage		2 (0.39)				2 (0.28)
Traumatic haemorrhage		1 (0.19)	1 (0.87)			2 (0.28)
Bleeding time abnormal		1 (0.19)	1 (0.87)			2 (0.28)
Arterial haemorrhage	1 (1.23)					1 (0.14)
Brain stem haemorrhage		1 (0.19)				1 (0.14)
Intracranial tumor haemorrhage	1 (1.23)					1 (0.14)
Intraventricular haemorrhage	1 (1.23)					1 (0.14)
Cerebellar haemorrhage	1 (1.23)					1 (0.14)
Subdural haemorrhage			1 (0.87)			1 (0.14)
Catheter site haemorrhage		1 (0.19)				1 (0.14)
Retinal haemorrhage			1 (0.87)			1 (0.14)
Vitreous haemorrhage		1 (0.19)				1 (0.14)
Duodenal ulcer haemorrhage	1 (1.23)					1 (0.14)
Anal haemorrhage		1 (0.19)				1 (0.14)
Ear haemorrhage		1 (0.19)				1 (0.14)
Lymph node haemorrhage			1 (0.87)			1 (0.14)
Mucocutaneous haemorrhage		1 (0.19)				1 (0.14)
Mucosal haemorrhage		1 (0.19)				1 (0.14)
Nail bed bleeding		1 (0.19)				1 (0.14)
Pelvic haemorrhage		1 (0.19)				1 (0.14)
Pericardial haemorrhage	1 (1.23)					1 (0.14)
Pulmonary alveolar haemorrhage	1 (1.23)					1 (0.14)
Pulmonary haemorrhage		1 (0.19)				1 (0.14)
Uterine haemorrhage	1 (1.23)					1 (0.14)
Vessel puncture site haemorrhage		1 (0.19)				1 (0.14)
Procedural haemorrhage		1 (0.19)				1 (0.14)

### 4.5 Outcomes due to haemorrhages

The prognosis of PARPis-associated haemorrhages was investigated by assessing the rate of outcomes after using PARPis. The outcomes include death, disability, hospitalization-initial or prolonged, life-threatening, and other serious medical events. As shown in **Table 5**, in all the cases of haemorrhages-related adverse events, olaparib took up 79 cases (12.44%), niraparib took up 477 cases (75.12%), rucaparib took up 72 cases (11.34%), talazoparib took up 5 cases (0.69%), and veliparib took up 2 cases (0.28%), respectively. The outcome of haemorrhages resulted in hospitalization in 31.81% patients. Specifically, in the cases of hospitalization, the proportion of niraparib (22.99%) was much higher than that of olaparib (4.72%), rucaparib (3.62%), talazoparib (0.32%), and veliparib (0.16%). Only niraparib caused disability (1.05%). In all the outcomes of patients with haemorrhages-related adverse events, the proportion of death was 4.72% (30/635). Of all death cases, olaparib accounted for 50%, niraparib 40%, rucaparib 6.67% and veliparib 3.33%. It may not mean that olaparib and niraparib are more dangerous, but most likely because olaparib and niraparib have been on the market longer, with more sales and more patients. Notably, though niraparib resulted in 38 cases (7.97%) life-threatening, olaparib resulted in more cases of death than niraparib (15, 18.99% vs 12, 2.52%).

**Table 5:** Clinical outcomes of patients with haemorrhages-related adverse events.

Outcome	Reports (N, %)				
	Olaparib	Niraparib	Rucaparib	Talazoparib	Veliparib
Death	15 (18.99)	12 (2.52)	2 (2.78)		1 (50)
Disability		5 (1.05)	0 (0.00)		
Hospitalization - initial or prolonged	30 (37.97)	146 (30.61)	23 (31.94)	2 (40)	1 (50)
Life-threatening	1 (1.27)	38 (7.97)	1 (1.39)		
Other serious (important medical event)	33 (41.77)	276 (57.86)	46 (63.89)	3 (60)	

### 4.6 Signal detection

The disproportionality results of ROR, PRR, IC and EBGM were shown in **Table 6**. The ROR (95% CI) for olaparib, rucaparib and talazoparib was 0.32 (0.26, 0.4), 0.43 (0.36, 0.52) and 0.3 (0.14, 0.68), respectively, which showed no significant signal. The ROR (95% CI) and IC (IC025) of niraparib were 1.13 (1.03, 1.12) and 0.17 (0.15), respectively, which shows significant association between niraparib and haemorrhage-related adverse events. For veliparib, although ROR (95% CI), PRR (χ2), and IC (IC025) showed significant signals, there were only two cases, which was not sufficient for algorithm calculations.

**Table 6:** Disproportionality analysis in FAERS database.

Drug	N	ROR (95% two-sided CI)	PRR (χ2)	IC (IC025)	EBGM (EBGM05)
Olaparib	88	0.32 (0.26, 0.4)	0.33 (123.99)	-1.6 (-)	0.33 (0.28)
Niraparib	517	1.13 (1.03, 1.23)*	1.12 (7.32)	0.17 (0.15)*	1.12 (1.04)
Rucaparib	112	0.43 (0.36, 0.52)	0.44 (81.99)	-1.18 (-)	0.44 (0.38)
Talazoparib	6	0.3 (0.14, 0.68)	0.31 (9.52)	-1.69 (-)	0.31 (0.16)
Veliparib	2	6.66 (1.41, 31.37)*	5.53 (7.7)*	2.47 (0.52)*	5.53 (1.51)

* Indicates a significant signal.

## 5 Discussion

PARPis are currently popular PARP-targeted drugs for precise treatment of patients with certain types of cancer, with or without defined BRCA mutations. In this study, we mined data from the FAERS database and analyzed the characteristics of haemorrhages cases related to olaparib, niraparib, rucaparib, talazoparib and veliparib. We found that haemorrhage-related adverse event was a matter of concern, which had not been reported or studied.

The mechanism underlying the interaction between PARPi and haemorrhage-related adverse events remains unclear, which may be due to long-time exposure to DNA damage. As known, PARPis can restrain DNA repair and induce the apoptosis of cancer cells by inhibiting PARP (28), with most functions performed by PARP-1 (85%–90%) and PARP-2 (10%–15%) (29). Studies have shown that thrombocytopenia is commonly encountered in the clinical use of PARPis (30, 31). Platelets are formed from mature megakaryocytes (MKs), and PARP1 is expressed in the megakaryocyte lineage with regulatory effects on hematopoietic stem cells. PARPis can reduce platelet formation by inhibiting PARP1 (32, 33), which may reduce the formation of platelets. As for the onset times of haemorrhage-related adverse events, PARPi-haemorrhage was characterized by an early onset, usually occurring within 6 months following initiation of therapy. While for niraparib, rucaparib and talazoparib, earlier onset times about one or two months should be mentioned, and thrombocytopenia of any grade is pronounced (34). As reported, PARPis-related thrombocytopenia typically occurred during the first month of treatment (35). The platelet count decreased remarkably during the first cycle after administering niraparib and rucaparib, and the concentrations plateaued after cycle 2 or 3 (35–37). This occurrence time was consistent with the onset time of haemorrhage and indicated that thrombocytopenia may be related to the haemorrhage adverse events.

The signal detection showed a signal with haemorrhage-related adverse reactions from niraparib, while olaparib, rucaparib, talazoparib did not produce a signal. Since veliparib is not on the market yet, there were only two haemorrhage-related adverse events from clinical study. Although the ROR (95% CI), PRR (χ2) and IC (IC025) for veliparib showed significant signals, it was difficult to pin down its meaning, and more clinical information was needed for pharmacovigilance analysis. A retrospective analysis of ovarian cancer patients treated with PARPis showed that patients in the niraparib group experienced more haemorrhage-related adverse reactions than olaparib, with 11 (35.5%), 20 (64.5%), and 18 (58.1%) bearing neutropenia, anemia, and thrombocytopenia, respectively (38). Thus, constant vigilance for the signs and symptoms of this toxicity is required when using PARPis, especially niraparib. The instructions for niraparib suggest that when platelet transfusion is needed for concentrations of platelets <100,000/mcL, the dose of PARPis should be reduced or discontinued. Thus, monitoring platelet counts during the first few months after using niraparib may help detect and preventhaemorrhage-related adverse events. Moreover, BRCA mutant patients are more likely to have severe adverse effects than BRCA wild-type patients. Therefore, BRCA mutant patients should be closely monitored for haemorrhages.

From the 725 cases of haemorrhage adverse events related to PARPis therapy, the main data sources of FAERS are European and American countries. North America has reported 615 cases (84.83%). Europe and Asia both have reported 50 cases (6.90%). This may be because the PARPis were initially marketed in America and Europe, as well as the data sources are mainly from North America. As for the five PARPis, olaparib and niraparib were involved in most cases. Olaparib was the first one on the market. However, niraparib, which was approved by FDA in 2017, seems more widely used. Since the dose frequency of niraparib is once daily, niraparib is more convenient and adaptable than olaparib. In addition, niraparib is more selective for PARP 1/2 than olaparib (39), indicating a higher treatment efficiency. The initial indications of olaparib and niraparib are ovarian cancer, fallopian tube, and primary peritoneal cancer, thus more than 79% patients are female. Last, multiple clinical trials have confirmed PARPis efficacy in BRCA mutated ovarian and breast cancer, as well as prostate, pancreatic cancer, and small cell lung cancer, irrespective of the BRCA status (40). These expanded indications may increase the male cases.

The cases of PARPi-related haemorrhage adverse events from FAERS dramatically increased from 2018 to 2021 (170 cases in 2018, 120 cases in 2019, 126 cases in 2020, 177 cases in 2021). However, the most cases were from niraparib (**Table 2** Reporting years) instead of olaparib. On May 8, 2020, the FDA expanded the indication of olaparib to include its combination with bevacizumab for first-line maintenance treatment of adult patients with advanced epithelial ovarian, fallopian tube, or primary peritoneal cancer who are in complete or partial response to first-line platinum-based chemotherapy and whose cancer is associated with homologous recombination deficiency positive status defined by either a deleterious or suspected deleterious BRCA mutation and/or genomic instability. The NCCN clinical practice guideline of Ovarian Cancer Including Fallopian Tube Cancer and Primary Peritoneal Cancer (Version 1. 2023) also recommends olaparib combination with bevacizumab as the first-line maintenance treatment of adult patients with advanced epithelial ovarian, fallopian tube, or primary peritoneal cancer. However, niraparib combination with bevacizumab for the treatment of the above cancers has not been approved by any official organization. Besides, niraparib is only approved for monotherapy. Therefore, the haemorrhage-related adverse events may not result from bevacizumab, although it is well-recognized in increasing the risk of serious bleeding in cancer patients.

It is well known that haemorrhage is a systemic pathological phenomenon. To understand the specific site of bleeding that may happen, we analyzed the PTs related to haemorrhages-related adverse events (**Table 4**). Among the identified PTs, petechiae, gingival bleeding, blood urine present, rectal haemorrhage, and vaginal haemorrhage are the top five PTs, which should be noted. It is difficult to determine whether these PTs are related to debulking surgery. Therefore, it is hard to say the post-operative complications could result in all these adverse events. As reported, gastrointestinal toxicities are common for all PARPis (41). The symptoms include nausea, abdominal pain, vomiting, diarrhea, constipation, dyspepsia, and stomatitis. Gastrointestinal haemorrhage is not mentioned. A phase trial of niraparib monotherapy of late-line treatment of ovarian cancer showed that 4 (1%) of 463 patients had grade 1-3 drug-related rectal haemorrhage and 1 death of drug-related gastric haemorrhage (18). In our study, the proportions of patients having rectal haemorrhage were 11.11% for olaparib (9/81), 7.35% for niraparib (38/517), and 6.96% for rucaparib (8/115), respectively. The proportions of patients having gastric haemorrhage were 1.23% for olaparib (1/81), 0.77 for niraparib (4/517), and 0.87% for rucaparib (1/115), respectively. Obviously, rectal haemorrhage should be monitored when using PARPis. In addition, the PTs of rectal haemorrhage together with gastrointestinal haemorrhage, gastric haemorrhage, haemorrhoidal haemorrhage, lower gastrointestinal haemorrhage, intestinal haemorrhage, large intestinal haemorrhage, upper gastrointestinal haemorrhage, anal fissure haemorrhage, oesophageal haemorrhage, duodenal ulcer haemorrhage, and anal haemorrhage occupy 116 cases (16.00%). These symptoms happen after using olaparib, niraparib and rucaparib. Thus, patients administered PARPis are warned of gastrointestinal haemorrhage, especially rectal haemorrhage.

In the pharmacovigilance analysis (**Table 5**), the proportion of death resulting from haemorrhage-related adverse events was 4.72%. This suggested a heightened awareness of this serious adverse effect. Five disability cases are all from niraparib. There was no significant difference in the proportion of life-threatening events among olaparib, rucaparib, talazoparib and veliparib. However, the risk of initial or prolonged hospitalization due to PARPi-haemorrhage appeared to be significantly higher in the niraparib group than in the other groups. As this was a real**-**world study that analyzed post**-**marketing surveillance data, the results should be more representative of real experience in clinical practice than those of clinical trials.

Although this study takes advantage of real-world research, there are some limitations to consider. First, FAERS is a spontaneous reporting system, to which either consumers or health-professional workers could report the adverse events. Thus, the reports from consumers may be misjudged. However, in this study, the adverse reaction of haemorrhage is quite easy for patients to judge. For example, the top five PTs (except for haemorrhage) are petechiae, gingival bleeding, blood urine present, rectal haemorrhage, and vaginal haemorrhage which are obvious symptoms to judge. Second, The FAERS data is mainly from America and Europe, which does not cover all the haemorrhage-related adverse events worldwide. The PARPis have been on the market for a short time so more clinical information needs to be collected. Third, because of the spontaneity of the FAERS reporting system, missing data exists, which may result in bias. However, the proportion of missing data in this study is less than 5% for important data, such as the indications for the use of PARPis (25/725), making less difference to the conclusions. Recently, Dhodapkar and colleagues analyzed 12 years of safety signals identified within the FAERS. They found that most of the potential safety signals found in FAERS led to the FDA’s regulatory actions. However, only one third of regulatory actions have been confirmed by published research, and none has been publicly evaluated by the Sentinel Initiative.

Their research emphasizes that a larger and more robust post-market safety study is needed to improve the quality of evidence and evaluate rare safety incidents (42), such as haemorrhage related adverse events with PARPis. Despite these limitations, the findings of this study indicate potential safety problems of haemorrhages when using PARPis.

## 6 Conclusion

Haemorrhage-related adverse events may seriously affect patient safety and tend to occur early. It is advised to pay close attention to tumor progression and take timely intervention measures when adverse drug reaction (ADR) or disease progression occurs so as to ensure safe and rational drug use. In this study, we provided a safety signal that haemorrhage-related adverse events should be monitored for when using niraparib. However, larger and more robust post-market safety studies are needed to improve the quality of this evidence. To our knowledge, this is the first pharmacovigilance analysis of haemorrhage-related adverse events associated with PARPis. Our study suggests a possible relationship between PARPis and haemorrhage-related adverse events in clinical practice.

## Data availability statement

Publicly available datasets were analyzed in this study. This data can be found here: FAERS database.

## Author contributions

All the authors were involved in the study. Study design and administration: ZJ, Extraction data: MG and PF, Analysis and interpretation of data: SW, Writing original draft: SW, Writing editing: MG and XZ. All authors contributed to the article and approved the submitted version.
